# Effects of exercise intervention on vascular endothelium functions of patients with impaired glucose tolerance during prediabetes mellitus

**DOI:** 10.3892/etm.2013.1064

**Published:** 2013-04-11

**Authors:** YIPING LIU, JIANWEI LI, ZHENGHONG ZHANG, YEDONG TANG, ZUOSONG CHEN, ZHENGCHAO WANG

**Affiliations:** 1 Laboratory of Sport Physiology and Biomedicine, School of Physical Education and Sport Sciences, Fuzhou, Fujian 350007;; 2 Provincial Key Laboratory for Developmental Biology and Neurosciences, College of Life Sciences, Fujian Normal University, Fuzhou, Fujian 350007;; 3 Department of Ultrasound, Fujian Provincial Hospital, Provincial Clinical College of Fujian Medical University, Fuzhou, Fujian 350001, P.R. China

**Keywords:** exercise intervention, impaired glucose tolerance, diabetes mellitus

## Abstract

Endothelial dysfunction (ED) is an early pathophysiological change in patients with impaired glucose tolerance (IGT) during prediabetes mellitus. This study was designed to test the hypothesis that exercise intervention contributes to the reversal of vascular endothelium-dependent dysfunction in middle-aged patients with IGT. Following exercise intervention, significant changes in endothelin (ET)-1, C-type natriuretic peptide (CNP), ΔDia-P, oral glucose tolerance test (OGTT)2h, fasting insulin, homeostasis model of assessment-insulin resistance (HOMA-IR), body fat percentage, waist circumference and waist to hip ratio were measured. However, no marked changes in carotid artery intima-media thickness (IMT), fasting blood glucose and BMI were observed following exercise intervention. Validity analysis of index changes in the two exercise intervention groups further confirmed there was no change. Exercise intervention increased CNP levels, decreased ET-1 levels and increased ΔDia-P, indicating improved vascular endothelium function. Decreased HOMA-IR following exercise suggests enhanced insulin sensitivity. Exercise intervention also improved glucose metabolism via decreased OGTT2h and fasting insulin. In addition, decreased waist circumference, ratio of waist to hip and body fat percentage following exercise intervention improved changes of body composition, including BMI, body fat and waist circumference. These results indicate that exercise intervention may reverse vascular endothelium-dependent dysfunction in middle-aged patients with IGT. This study also provided direct clinical data supporting the use of exercise intervention to prevent diabetes mellitus (DM) during the early stage.

## Introduction

Impaired glucose tolerance (IGT) is an early characteristic of prediabetes mellitus, in addition to the elevated postprandial glucose (1,2). Insulin resistance (IR) is often detected in patients with glucose metabolism disorders (3). IR is the compensatory excessive secretion of insulin for maintaining normal physiological functions and subsequently results in hyperinsulinemia (3). In general, the important factors affecting IR (1) include being overweight or obese, low levels of physical exercise and genetic factors (2). Currently, the physically less active modern lifestyle is a significant factor leading to the increased incidence of IGT and diabetes mellitus (DM) with IR (4–6).

IR may coexist with hepatic resistance leading to increases of glycogen production and output, and postprandial hyperglycemia. IR may also coexist with muscular resistance leading to the reduction of insulin-stimulated glucose uptake or manifestations and of dystrophin-glycoprotein generation/storage. Finally, the insulin suppression of lipolysis is attenuated (2,7,8). In the 1980s, an early study concerning the prevention of diabetes in individuals with IGT was performed in Daqing, China (9). Subsequently, several diabetes prevention studies, including a diabetes prevention study (DPS) in Finland (10) and a diabetes prevention program (DPP) in the United States of America (11) showed that the incidence of diabetes was significantly reduced via lifestyle intervention during IGT. Therefore, intervention during IGT may be an important method for the prevention of DM.

By contrast, the risk of cardiovascular disease (CVD) is similar to that of DM in patients with IGT (12,13). Therefore, the main harmful effect of IGT is not only the transformation to diabetes, but also the increased risk of CVD (13). IGT is also an important indicator of CVD in patients with endothelial dysfunction (ED) as the early important pathophysiological marker (14). ED in functional performance is the attenuated resilience and vasodilation of large vessels (14). ED is also strongly correlated with obesity and IR. IR is a vascular complication of DM and plays an important role in CVD pathogenesis (3,13,14). Therefore, it is extremely important to prevent DM cardiovascular complications and to reverse ED via early IGT intervention.

At present, exercise is the best treatment for metabolic syndrome, and has remarkable effects on the prevention of DM and CVD with IGT (5,6). Previous studies indicated that exercise increases energy consumption, reduces body fat, increases muscle mass and also has a profound effect on the endocrine system (4–6,11). Compared with other methods for controlling IGT, exercise therapy is a low cost, simple, properly implemented and safe strategy without side-effects. These advantages have attracted much attention in recent years (4–6,11). In addition, the population affected by DM pathogenesis has gradually become younger (2,4,8,10), which increasingly affects the working population at its prime age. It is necessary to further investigate the effects of exercise on middle-aged patients with IGT and IR in order to provide a clinical basis for exercise in the prevention of DM.

Walking is one of the most simple and commonly used fitness exercises, and resistance exercise has gained increasing attention since it is able to increase the basal metabolic rate, facilitate energy consumption and thereby increase insulin sensitivity (5,6,11). In the current study, two different exercise interventions (moderate intensity walking exercise and walking plus resistance exercise) were used for 24 weeks. The serum physiological and biochemical indices and vascular endothelial function changes were monitored in the middle-aged patients with IGT. The vascular endothelial function-improving effects of exercise interventions were studied, which provide a clinical and theoretical basis for the prevention of early DM.

## Subjects and methods

### Study subjects and groups

In this study, 61 patients with IGT (mean age 49.8±4.8 years) were selected based on medical screening from two residential communities (the Cangshan and Gushan Districts in Fuzhou, the capital city of Fujian Province, China). The patients had no cardiopulmonary or systematic diseases affecting fitness to exercise. Patients from a non-sport orientated population undertook a 24 week-long course of fitness exercises. The IGT participants were randomly divided into three groups: the control group without exercise, walking exercise group and walking plus resistance exercise group. This study followed International Journal of Sports Medicine’s (IJSM’s) ethical standards (15) and was approved by the Institutional Review Board of Fujian Normal University and Fujian Medical University (Fuzhou, China). All participants signed a written informed consent document.

### Exercise models and methods

Participants in the control group did not participate in exercise. In the walking exercise group, participants were requested to look straight ahead and keep their stomach in, chest out, head out and shoulders back, but relax and swing their arms rhythmically as they walked. The exercise intensity was at 60∼70% maximum heart rate (HR)_max_, 4 times a week for 60 min each time, including 5 min stretching and warming-up, 50 min walking exercise and 5 min relaxation. The walking exercise was performed on a running machine. The exercise intensity started from 50–60% HR_max_, and then gradually increased to 60–70% HR_max_.

For the walking plus resistance exercise group, the exercise included 30 min resistance exercise following 20 min walking exercise. The resistance exercise focused on weight reduction, and included upper arm, chest and waist exercises (15 pulls on the lateral pulldown device with 2–3 repeats and 15 straight-arm forwards using the abdominal machine with 3–4 repeats), abdominal exercises (15–20 sit-ups on the sit-up apparatus with 2–3 repeats and 15–20 presses on the leg press machine with 2–3 repeats) and leg exercises (15 leg presses on the leg press machine with 2–3 repeats and 15 extensions on the leg extension machine with 2–3 repeats).

### Implementation and monitoring of exercise

Adaptive training was implemented for one week prior to the formal exercises. The number of heart beats within 10 sec (HR_10sec_) at the different stages was recorded during the exercise process. The HR was calculated as six times HR_10sec_. The exercise intensity was adjusted and controlled based on self-reporting and the body reactions of the participants. Physiological and biochemical indices were detected before and after the whole exercise intervention.

### Physiological and biochemical indices

Morphological parameters were measured using a body composition resistance measurement instrument Biospace InBody 3.0 (Biospace Co. Ltd. Soul, South-Korea) according to the national physiological constitution monitoring requirements, which included BMI, body fat percentage and other variables (16). The biochemical indices were detected with a variety of kits. The oral glucose tolerance test (OGTT) was performed for the diagnosis of normal glucose tolerance (NGT) and IGT according to the guidelines of 2004 China diabetes prevention and control (17). Homeostasis model assessment (HOMA): HOMA-IR = fasting plasma insulin concentration (FINS, mIU/l) x fasting blood glucose (FBG, mmol/l)/22.5. Brachial artery endothelium dependent vasodilation was measured using color Doppler supersonic diagnostic equipment (IU-22; Phillips-Medisize Corporation, Hudson, WI, USA) with a L17-5 transducer of 17 to 5 MHz extended frequency range. Brachial artery endothelium-dependent vasodilation: ΔDia-P% = (maximum diameter inside brachial artery with 1 min decompression − basic diameter)/basic diameter ×100.

### Statistical analysis

All experimental data are presented as mean ± SD. The differences in mean values between multiple groups were evaluated using a two-way ANOVA. A paired Student’s t-test was used to evaluate statistical differences due to the intervention. P<0.05 was considered to indicate a statistically significant result and P<0.01 was considered to indicate a notable statistical significance.

## Results

### Equilibrium analysis of vascular endothelium functions prior to exercise intervention among the three groups

Fifty patients with IGT completed the whole experiment out of 61 patients who began the study. Four from the control group, three from the walking group and four from the walking plus resistance group did not complete the experiment; the drop out rate was 18.03%. There were no significant differences (P>0.05) in each index of vascular endothelium function among the three groups prior to the exercise intervention (Table I). No significant differences (P>0.05) were observed in FBG, OGTT2h, fasting insulin and HOMA-IR prior to the exercise intervention among the three groups (Table II). No significant differences (P>0.05) were observed in BMI, body fat percentage, waist circumference and waist to hip ratio prior to exercise intervention among the three groups (Table III).

### Comparison of all indices prior to and following exercise intervention

The comparison of indices showed no significant differences (P>0.05) prior to and following exercise interference in the control group, with the exception of FBG (P<0.05; Table IV). The comparison of indices showed there were significant differences (P<0.05) for C-type natriuretic peptide (CNP), endothelin (ET)-1, ΔDia-P, OGTT2h, fasting insulin, HOMA-IR, body fat percentage, waist circumference and waist to hip ratio prior to and following exercise intervention in the walking exercise group, however, FBG, carotid artery intima-media thickness (IMT) and BMI were not significantly different (P>0.05; Table V).

A comparison of indices prior to and following exercise intervention in the walking plus resistance exercise group was performed. The comparison of all indices showed there were significant differences (P<0.05) for CNP, ET-1, ΔDia-P, OGTT2h, fasting insulin, HOMA-IR, body fat percentage, waist circumference and waist to hip ratio prior to and following exercise intervention in the walking plus resistance group, but no significant differences in FBG, carotid artery IMT and BMI (P>0.05; Table VI).

The effect of exercise on brachial endothelium-dependent vasodilation was also assessed. Brachial artery endothelium-dependent vasodilation was improved by exercise intervention in one patient. Brachial artery diameters measured at three randomly selected sites by ultrasound were 0.440, 0.443 and 0.450 cm prior to exercise intervention. However, following intervention the diameters measured at these three sites were 0.514, 0.501 and 0.483 cm, respectively. These results indicate that brachial artery vasodilation may be improved by the exercise intervention.

### Effect on vascular endothelium functions following exercise intervention

Variance analysis of vascular endothelium functions showed that exercise intervention significantly increased CNP levels (P<0.05; Table VII) compared with those of the control group. The CNP level in the walking plus resistance exercise group was further increased compared with that in the walking group (P<0.05; Table VII). Exercise intervention markedly decreased ET-1 levels compared with those in the control group. In addition, the ET-1 level in the walking plus resistance exercise group was significantly lower than that in the walking group (P<0.05, Table VII). Significant changes in the percentage changes in artery diameter following a 5-min compression (ΔDia-P) were observed following exercise intervention (P<0.05; Table VII), but no significant changes were observed between the two exercise intervention groups (P>0.05; Table VII).

### Effect analysis of HOMA-IR and glucose metabolism following exercise intervention

Variance analysis of HOMA-IR and glucose metabolism showed that exercise intervention significantly decreased OGTT2h, fasting insulin and HOMA-IR levels (P<0.05; Table VIII) but not the FBG level (P>0.05; Table VIII). However, there were no marked changes between the two exercise intervention groups (P>0.05, Table VIII).

### Effect analysis of body composition following exercise intervention

Variance analysis of body composition demonstrated that the exercise intervention significantly increased body fat percentage and the waist to hip ratio (P<0.05; Table IX). However, there were no significant differences between the two exercise intervention groups (P>0.05; Table IX). Marked changes of waist circumference were observed among the three groups (P<0.05; Table IX), while there were no significant differences between the two exercise intervention groups (P>0.05; Table IX).

## Discussion

Numerous studies have demonstrated that lifestyle intervention is able to significantly decrease the occurrence of DM in the population with IGT, and that IGT is a critical stage at which to prevent DM (9,12,13). Our study clearly demonstrated that exercise intervention significantly improved endothelium-dependent vascular functions through enhancing the physiological constitution, increasing insulin sensitivity and preventing further vascular deterioration during prediabetes mellitus.

The patients with IGT may be considered as sub-healthy, since IGT may transform into DM and increase the CVD risk (9,12,13). Dysfunction of the vascular endothelium is closely correlated with CVD (3,13) and is also a key link of DM complications (14). Usually, patients with IGT do not have clear clinical symptoms and do not receive sufficient attention. We used outpatient and physical examination records of local residents to enhance the detection of patients with IGT. The results showed that IGT may be the last key stage at which to prevent DM. To understand the possible mechanism of the transformation of IGT into DM, patients with IGT were randomly divided into three groups with different exercise interventions in order to investigate the DM preventive effects. Epidemiological surveys have shown that lack of exercise is closely correlated with CVD and appropriate exercise is an important method for reducing the risk of CVD by improving endothelial functions (11,18,19). Therefore, the effects of two different types of exercise on endothelium-dependent vasodilation were examined in patients with IGT. In general, fitness exercise included aerobic and resistance exercise. Aerobic exercise may increase the lipoprotein lipase (LPL) activity and promote lipolysis *in vivo* leading to the consumption of more fat but less glycogen with improved vascular endothelial functions in patients with CVD (6,19). By contrast, the most significant feature of resistance exercise is the improvement of skeletal muscle quality and the enhancement of cardiovascular endurance (20,21). Resistance exercise increases fat oxidation, plasma catecholamine levels and excess post-exercise oxygen consumption, and improves glucose metabolism levels (5,6,20,21). Previous studies indicated that aerobic exercise may improve the vascular endothelial function of patients with coronary diseases (19), while resistance exercise may also improve the symptoms of CVD (21).

A number of studies have demonstrated that exercise may improve insulin sensitivity by increasing energy consumption (5,6,9). In the present study, we demonstrated that exercise improved glucose metabolism in middle-aged patients with IGT and caused significant differences in BMI, body fat percentage, waist circumference, waist to hip ratio, OGTT2h blood glucose, fasting insulin and HOMA-IR prior to and following exercise intervention. These results indicate that exercise intervention may enhance insulin sensitivity, which is further supported by the findings of Smutok *et al*(8) and Melby *et al*(21). Notably, increased nitric oxide (NO) levels and decreased IR were observed in IR rats during exercise experiments, suggesting that exercise may promote the release of vasodilatory substances and enhance insulin receptor sensitivity (19,22). ED plays an important role in the pathogenesis of diabetic vascular complications and CVD (14,22), since vascular endothelial cells (ECs) are able to synthesize and secrete a variety of bioactive factors, including ET-1 and CNP (22,23). ET-1 is a small vasoactive polypeptide that has the strongest effect and the longest duration of action as a vasoconstrictor via binding to the ET receptor *in vivo*(23,24). CNP is another vasoactive peptide that inhibits the proliferation and migration of vascular smooth muscle cells and extracellular matrix formation, in addition to acting as a vasodilator (23,24). The results of the present study have demonstrated that ET-1 levels were reduced but CNP levels increased following exercise intervention. Exercise intervention improved endothelial functions in middle-aged patients with IGT, particularly in the walking plus resistance exercise group.

In conclusion, exercise intervention improved endothelium-dependent vasodilation in middle-aged patients with IGT. Walking and resistance exercise improved the body composition of the patients, reversed IR and increased the ability of insulin to bind with its receptor in the target organs (23). Furthermore, exercise reduces OGTT2h glucose changes and improves glucose tolerance (13). In the current study, the effects of physical exercise intervention provided direct clinical evidence of the effectiveness of this approach for preventing early DM in patients with IGT.

## Figures and Tables

**Table I:** Analysis of vascular endothelium functions prior to exercise intervention in patients with IGT.

Group	n	CNP (pg/ml)	ET-1 (pg/ml)	Carotid artery IMT (mm)	ΔDia-P (%)
Control	21	14.553±3.296	140.829±32.379	1.056±0.181	10.829±1.597
Walking exercise	20	14.099±2.283	141.955±32.797	1.051±0.206	10.853±1.634
Walking + resistance exercise	20	14.736±4.561	148.756±39.149	1.039±0.206	10.493±1.533
F		0.019	0.306	0.038	0.324
P-value		0.982	0.737	0.963	0.724

P<0.05 was considered to indicate a statistically significant result among the three groups. IGT, impaired glucose tolerance; CNP, C-type natriuretic peptide; ET-1, endothelin-1; IMT, intima-media thickness; ΔDia-P, the percentage changes in artery diameter after a 5-min compression.

**Table II:** Analysis of HOMA-IR and glucose metabolism prior to exercise intervention in patients with IGT.

Group	n	FBG (mmol/l)	OGTT2h (mmol/l)	Fasting insulin (mIU/l)	HOMA-IR
Control	21	5.771±0.310	9.352±0.623	27.018±8.009	6.967±2.279
Walking exercise	20	5.670±0.264	9.345±0.575	26.747±8.022	6.948±2.288
Walking + resistance exercise	20	5.755±0.265	9.345±0.568	26.936±7.869	6.981±2.241
F		0.761	0.001	0.006	0.001
P-value		0.472	0.999	0.994	0.999

P<0.05 was considered to indicate a statistically significant result among the three groups. IGT, impaired glucose tolerance; FBG, fasting blood glucose; OGTT, oral glucose tolerance test; HOMA, homeostasis model assessment; IR, insulin resistance.

**Table III:** Analysis of body composition prior to exercise intervention in patients with IGT.

Group	n	BMI (kg/m^2^)	Body fat (%)	Waist circumference (cm)	Waist to hip ratio
Control group	21	27.211±1.296	25.367±2.140	91.229±4.791	0.899±0.028
Walking exercise	20	27.100±1.283	25.415±1.945	90.990±4.777	0.897±0.027
Walking + resistance exercise	20	26.523±1.259	25.630±1.272	89.161±4.269	0.898±0.023
F		1.691	0.119	1.212	0.035
P-value		0.193	0.888	0.305	0.966

P<0.05 was considered to indicate a statistically significant result among the three groups. IGT, impaired glucose tolerance.

**Table IV:** Comparison of all indices before and after the exercise intervention in the control group.

Index	Before intervention	After intervention	t	P-value
ET-1 (pg/ml)	140.829±32.380	143.125±40.424	0.719	>0.05
CNP (pg/ml)	14.553±4.675	14.397±4.536	0.797	>0.05
ΔDia-P (%)	10.830±1.597	10.326±1.799	0.471	>0.05
Carotid artery IMT (mm)	1.056±0.188	1.054±0.189	0.227	>0.05
Fasting blood glucose (mmol/l)	5.771±0.310	5.676±0.259	2.197	<0.05
OGTT2h (mmol/l)	9.352±0.623	9.371±0.574	0.677	>0.05
Fasting insulin (mIU/l)	27.018±8.009	26.999±7.904	0.247	>0.05
HOMA-IR	6.967±2.279	7.010±2.249	0.289	>0.05
BMI (kg/m^2^)	27.211±1.296	27.162±1.283	1.214	>0.05
Body fat percent (%)	25.367±2.140	25.314±1.951	0.845	>0.05
Waist circumference (cm)	91.229±4.791	91.239±4.794	0.279	>0.05
Waist to hip ratio	0.899±0.028	0.898±0.027	1.595	>0.05

P<0.05 was considered to indicate a statistically significant result. CNP, C-type natriuretic peptide; ET-1, endothelin-1; IMT, intima-media thickness; ΔDia-P, the percentage changes in artery diameter after a 5-min compression; OGTT, oral glucose tolerance test; HOMA, homeostasis model assessment; IR, insulin resistance.

**Table V:** Comparison of all indices before and after exercise intervention in the walking exercise group.

Index	Before intervention	After intervention	t	P-value
ET-1 (pg/ml)	141.955±32.797	148.006±34.183	4.389	<0.01
CNP (pg/ml)	14.825±4.622	16.275±4.691	11.747	<0.01
ΔDia-P (%)	10.853±1.635	12.653±1.705	2.233	<0.05
Carotid artery IMT (mm)	1.051±0.197	1.048±0.203	1.314	>0.05
Fasting blood glucose (mmol/l)	5.670±0.564	5.585±0.528	1.091	>0.05
OGTT2h (mmol/l)	9.345±0.575	8.825±0.434	7.218	<0.01
Fasting insulin (mIU/l)	26.747±8.022	25.022±6.742	5.435	<0.01
HOMA-IR	6.948±2.288	6.224±2.019	7.857	<0.01
BMI (kg/m^2^)	27.138±1.749	26.332±1.779	1.443	>0.05
Body fat percent (%)	25.415±1.945	23.850±2.118	9.457	<0.01
Waist circumference (cm)	90.990±4.777	88.313±3.828	3.535	<0.01
Waist to hip ratio	0.897±0.027	0.879±0.025	11.307	<0.01

P<0.05 was considered to indicate a statistically significant result. CNP, C-type natriuretic peptide; ET-1, endothelin-1; IMT, intima-media thickness; ΔDia-P, the percentage changes in artery diameter after a 5-min compression; OGTT, oral glucose tolerance test; HOMA, homeostasis model assessment; IR, insulin resistance.

**Table VI:** Comparison of all indices before and after exercise intervention in the walking plus resistance exercise group.

Index	Before intervention	After intervention	t	P-value
ET-1 (pg/ml)	148.756±39.149	141.947±36.550	2.492	<0.05
CNP (pg/ml)	14.736±4.561	17.015±4.623	−9.778	<0.01
ΔDia-P (%)	10.493±1.533	13.196±2.065	2.333	<0.05
Carotid artery IMT (mm)	1.039±0.206	1.032±0.207	1.896	>0.05
Fasting blood glucose (mmol/l)	5.755±0.565	5.600±0.515	1.808	>0.05
OGTT2h (mmol/l)	9.345±0.568	8.675±0.463	11.719	<0.01
Fasting insulin (mIU/l)	26.936±7.869	24.967±6.922	7.855	<0.01
HOMA-IR	6.981±2.241	6.209±2.035	8.680	<0.01
BMI (kg/m^2^)	26.523±1.359	25.978±1.286	1.252	>0.05
Body fat percent (%)	25.630±1.272	23.855±1.008	11.335	<0.01
Waist circumference (cm)	89.161±4.270	87.847±3.045	2.443	<0.05
Waist to hip ratio	0.898±0.023	0.884±0.019	7.590	<0.01

P<0.05 was considered to indicate a statistically significant result. CNP, C-type natriuretic peptide; ET-1, endothelin-1; IMT, intima-media thickness; ΔDia-P, the percentage changes in artery diameter after a 5-min compression; OGTT, oral glucose tolerance test; HOMA, homeostasis model assessment; IR, insulin resistance.

**Table VII:** Analysis of vascular endothelium functions following exercise intervention in patients with IGT.

Group	n	CNP (pg/ml)	ET-1 (pg/ml)	Carotid artery IMT (mm)	ΔDia-P (%)
Control group	17	0.156±0.898	−2.296±14.635	0.002±0.029	0.004±0.037
Walking exercise	17	−1.450±0.552^b^	−6.051±6.165	0.003±0.019	−0.800±1.602
Walking exercise + resistance exercise	16	−2.280±1.043^b,c^	−6.810±12.221^a,c^	0.007±0.011	−1.104±2.116^a^
F		43.003	6.479	0.508	2.924
P-value		0.000	0.003	0.605	0.062

a P<0.05 vs. control group;

b P<0.01 vs. control group;

c P<0.01 vs. walking exercise group. IGT, impaired glucose tolerance; CNP, C-type natriuretic peptide; ET-1, endothelin-1; IMT, intima-media thickness; ΔDia-P, the percentage changes in artery diameter after a 5-min compression.

**Table VIII:** Analysis of HOMA-IR and glucose metabolism following exercise intervention in IGT patients.

Group	n	FBG (mmol/l)	OGTT2h (mmol/l)	Fasting insulin (mIU/l)	HOMA-IR
Control group	17	0.095±0.199	−0.019±0.129	0.019±0.353	−0.043±0.681
Walking exercise	17	0.085±0.075	0.520±0.322^a^	1.725±1.419^a^	0.724±0.412^a^
Walking exercise + resistance exercise	16	0.155±0.182	0.670±0.256^a^	1.970±1.121^a^	0.772±0.398^a^
F		1.093	44.299	20.911	16.159
P-value		0.342	0.000	0.000	0.000

a P<0.01 vs. control group. IGT, impaired glucose tolerance; FBG, fasting blood glucose; OGTT, oral glucose tolerance test; HOMA, homeostasis model assessment; IR, insulin resistance.

**Table IX:** Analysis of body composition following exercise intervention in patients with IGT.

Group	n	BMI (kg/m^2^)	Body fat (%)	Waist circumference (cm)	Waist to hip ratio
Control group	17	0.050±0.187	0.052±0.284	−0.010±0.164	0.001±0.003
Walking exercise	17	0.305±0.327	1.565±0.740^a^	2.963±3.749^a^	0.018±0.007^a^
Walking + resistance exercise	16	0.345±0.482	1.775±0.701^a^	1.314±2.404^b^	0.014±0.008^a^
F		1.622	49.577	6.975	37.442
P-value		0.310	0.000	0.002	0.000

a P<0.01 vs. control group.

b P<0.05 vs. walking exercise group. IGT, impaired glucose tolerance.
